# DGIdb 5.0: rebuilding the drug–gene interaction database for precision medicine and drug discovery platforms

**DOI:** 10.1093/nar/gkad1040

**Published:** 2023-11-11

**Authors:** Matthew Cannon, James Stevenson, Kathryn Stahl, Rohit Basu, Adam Coffman, Susanna Kiwala, Joshua F McMichael, Kori Kuzma, Dorian Morrissey, Kelsy Cotto, Elaine R Mardis, Obi L Griffith, Malachi Griffith, Alex H Wagner

**Affiliations:** Steve and Cindy Rasmussen Institute for Genomic Medicine, Nationwide Children’s Hospital, Columbus, OH 43205, USA; Steve and Cindy Rasmussen Institute for Genomic Medicine, Nationwide Children’s Hospital, Columbus, OH 43205, USA; Steve and Cindy Rasmussen Institute for Genomic Medicine, Nationwide Children’s Hospital, Columbus, OH 43205, USA; Steve and Cindy Rasmussen Institute for Genomic Medicine, Nationwide Children’s Hospital, Columbus, OH 43205, USA; Department of Medicine, Washington University, St Louis, MO 63110, USA; Department of Medicine, Washington University, St Louis, MO 63110, USA; Department of Medicine, Washington University, St Louis, MO 63110, USA; Steve and Cindy Rasmussen Institute for Genomic Medicine, Nationwide Children’s Hospital, Columbus, OH 43205, USA; Department of Medicine, Washington University, St Louis, MO 63110, USA; Department of Medicine, Washington University, St Louis, MO 63110, USA; Steve and Cindy Rasmussen Institute for Genomic Medicine, Nationwide Children’s Hospital, Columbus, OH 43205, USA; Department of Pediatrics, The Ohio State University College of Medicine, Columbus, OH 43210, USA; Department of Medicine, Washington University, St Louis, MO 63110, USA; Department of Medicine, Washington University, St Louis, MO 63110, USA; Steve and Cindy Rasmussen Institute for Genomic Medicine, Nationwide Children’s Hospital, Columbus, OH 43205, USA; Department of Pediatrics, The Ohio State University College of Medicine, Columbus, OH 43210, USA

## Abstract

The Drug–Gene Interaction Database (DGIdb, https://dgidb.org) is a publicly accessible resource that aggregates genes or gene products, drugs and drug–gene interaction records to drive hypothesis generation and discovery for clinicians and researchers. DGIdb 5.0 is the latest release and includes substantial architectural and functional updates to support integration into clinical and drug discovery pipelines. The DGIdb service architecture has been split into separate client and server applications, enabling consistent data access for users of both the application programming interface (API) and web interface. The new interface was developed in ReactJS, and includes dynamic visualizations and consistency in the display of user interface elements. A GraphQL API has been added to support customizable queries for all drugs, genes, annotations and associated data. Updated documentation provides users with example queries and detailed usage instructions for these new features. In addition, six sources have been added and many existing sources have been updated. Newly added sources include ChemIDplus, HemOnc, NCIt (National Cancer Institute Thesaurus), Drugs@FDA, HGNC (HUGO Gene Nomenclature Committee) and RxNorm. These new sources have been incorporated into DGIdb to provide additional records and enhance annotations of regulatory approval status for therapeutics. Methods for grouping drugs and genes have been expanded upon and developed as independent modular normalizers during import. The updates to these sources and grouping methods have resulted in an improvement in FAIR (findability, accessibility, interoperability and reusability) data representation in DGIdb.

## Graphical Abstract

**Graphical Abstract:**
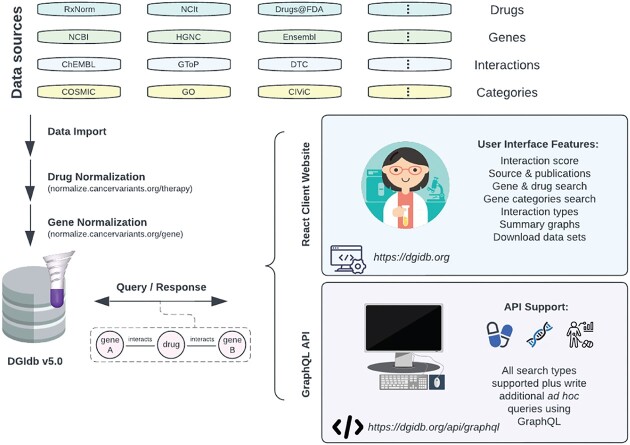

## Introduction

First released in 2013 (1), the Drug–Gene Interaction Database (DGIdb, https://dgidb.org) aggregates drugs, genes and their interactions into a single, computable resource. The primary goal of DGIdb since release has been to make the druggable genome (2,3) accessible for researchers seeking to generate new hypotheses for genome-wide data sets. By being able to identify the set of drugs that interact with genes or gene products of interest, DGIdb enables unique insights for potential therapeutic benefits in interactions that might not otherwise be visible. To accomplish this goal, DGIdb imports drug and gene claims from disparate sources and applies a rigorous grouping approach to match claims to their corresponding therapeutic or genomic concepts. Additional databases and literature sources are then mined to associate observed (or inferred) interactions between normalized drugs and genes. By aggregating and harmonizing data from multiple disparate resources, DGIdb has provided a toolkit for broad evaluation of the potential druggability of a gene or gene set.

Since its first release, DGIdb has undergone numerous updates to its feature set to improve service functionality, data quality and available tooling. DGIdb 2.0 (2016) (4) saw the introduction of a REST application programming interface (API) and grouping methods to improve harmonization of imported drug and gene claims, while DGIdb 3.0 (2018) (5) substantially updated existing and new sources to the resource (while also iterating further on the API). The last release, DGIdb 4.0 (2021) (6), focused on integration with crowdsource efforts and saw the introduction of community-contributed data sources such as the Drug Target Commons (7) and Wikidata (8).

Here, we describe DGIdb version 5.0 and our updates to enhance its utility in clinical research applications and drug discovery pipelines. As part of this major version release, we have completely refactored DGIdb into separate client and server components to create consistency of data access across the API and web interface. The new web application provides dynamic data visualizations and consistency in the display of user interface (UI) elements. The DGIdb database and web server have modernized existing import methods to accommodate changes in the available data from previously imported services. To support workflows with different data requirements, we have replaced our existing RESTful API endpoints with a central GraphQL API. This new endpoint supports *ad hoc* queries using the GraphQL query language for customized retrieval of drugs, genes and gene category annotations (as well as all associated sources, publications and additional data points) from DGIdb. By utilizing our new GraphQL endpoint, users with differing data needs can now access data with greater precision and efficiency. DGIdb has also been expanded to include six new sources for drug and gene records, and six existing sources have been updated to their latest version. Some of the new sources, such as Drugs@FDA, have been added to specifically enhance annotations of regulatory approval to existing drug–gene interaction claims. Lastly, we describe updates to our grouping methods for drugs and genes as independent, modular normalizers for improving the findability, accessibility, interoperability and reusability (9) of data within DGIdb.

## New features and enhancements

### Application framework updates (Ruby, React, GraphQL)

**Figure 1.**
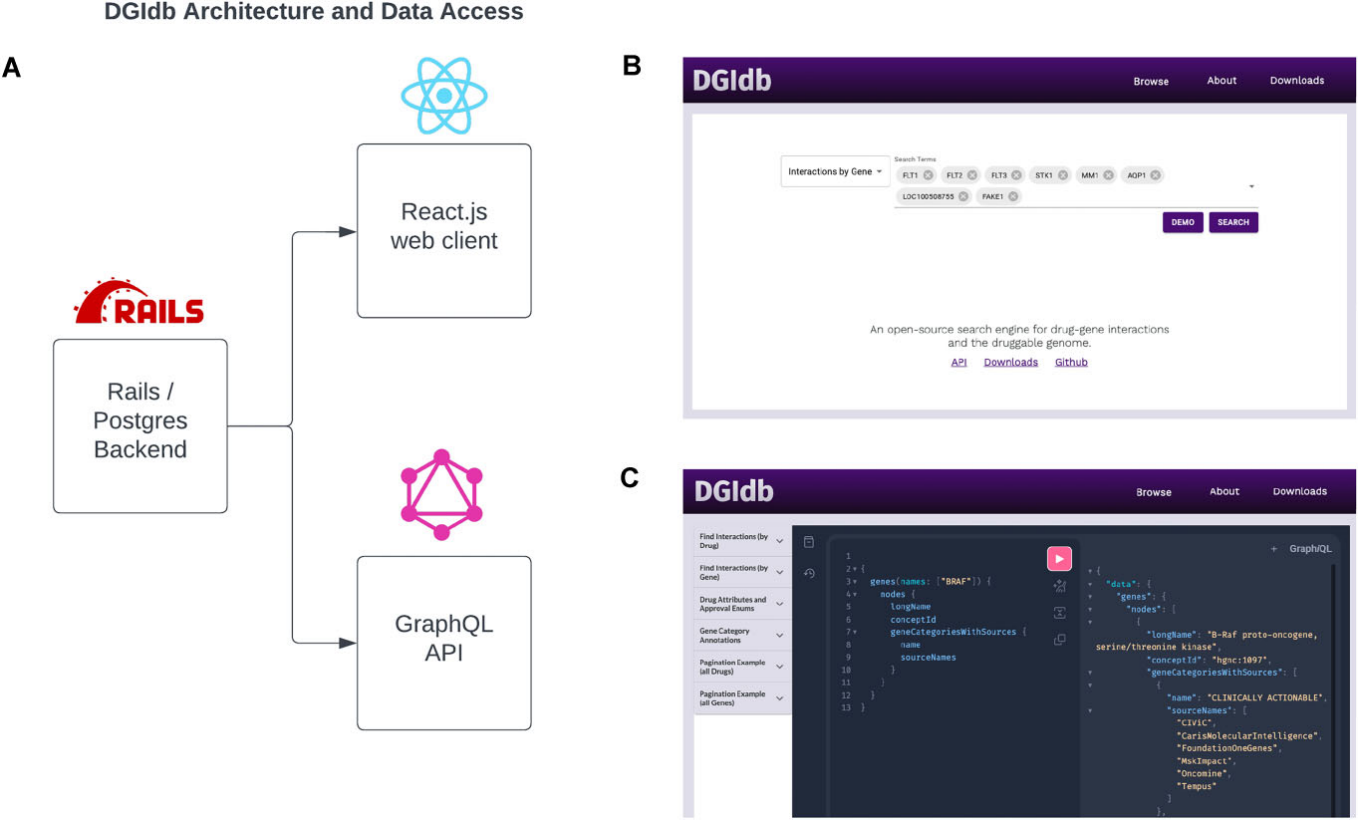
Architecture and data access for DGIdb v5.0. (**A**) DGIdb v5.0’s data are housed in a PostgreSQL database supported by Ruby on Rails version 6.1.3. Data from DGIdb is accessible via an HTML GUI built with React.js (http://dgidb.org) or through a GraphQL API (https://dgidb.org/api/graphql). (**B**) DGIdb’s web interface has been redesigned using the React.js framework. All previous functions and search types (interactions by drug, interactions by gene, gene categories) are accessible through the new interface (http://dgidb.org). (**C**) DGIdb supports GraphQL queries through the new API page. Queries can be sent here via API calls (https://dgidb.org/api/graphql) or sent manually through our GraphQL playground widget. Further documentation and example queries can be found at http://dgidb.org/api.

The key focus of our version 5.0 update was to rebuild the platform for incorporation into clinical research applications and drug discovery pipelines. Past versions of DGIdb have been built exclusively using the Ruby on Rails platform overlaying a PostgreSQL database. In this update, we have split DGIdb into separate client and server components with the goal of achieving greater flexibility in database implementation and the end user experience (Figure 1). The DGIdb server is still implemented with Ruby on Rails but now utilizes a GraphQL API to interface with a PostgreSQL database. This is in contrast to past versions of DGIdb that employed a traditional model–view–controller architecture (Figure 1A). As a consequence of these updates, many existing data importers have been modified or rewritten, and additional GraphQL-specific data model definitions have been added.

The client component of DGIdb has been rebuilt into a separate ReactJS interface with two primary methods for accessing the underlying data (Figure 1B and C). The web interface has been completely redesigned under this framework to streamline data access within DGIdb for clinical and drug discovery data analysis pipelines (Figure 1B). In addition to supporting this new web interface, the separation of client/server components has allowed migration to a GraphQL-based API for DGIdb (Figure 1C). This new endpoint utilizes the GraphQL query language to exploit node–edge relationships between data within DGIdb, allowing users to write customized, *ad hoc* queries for data points of interest. While the previous REST architecture restricted the user to predefined searches, GraphQL allows users to perform more flexible, semantically defined queries to fit the data requirements of individual applications. These formatted queries can be sent through our open API (https://dgidb.org/api/graphql). More details on these changes, as well as information on example queries, can be found below.

### New and updated sources

**Figure 2.**
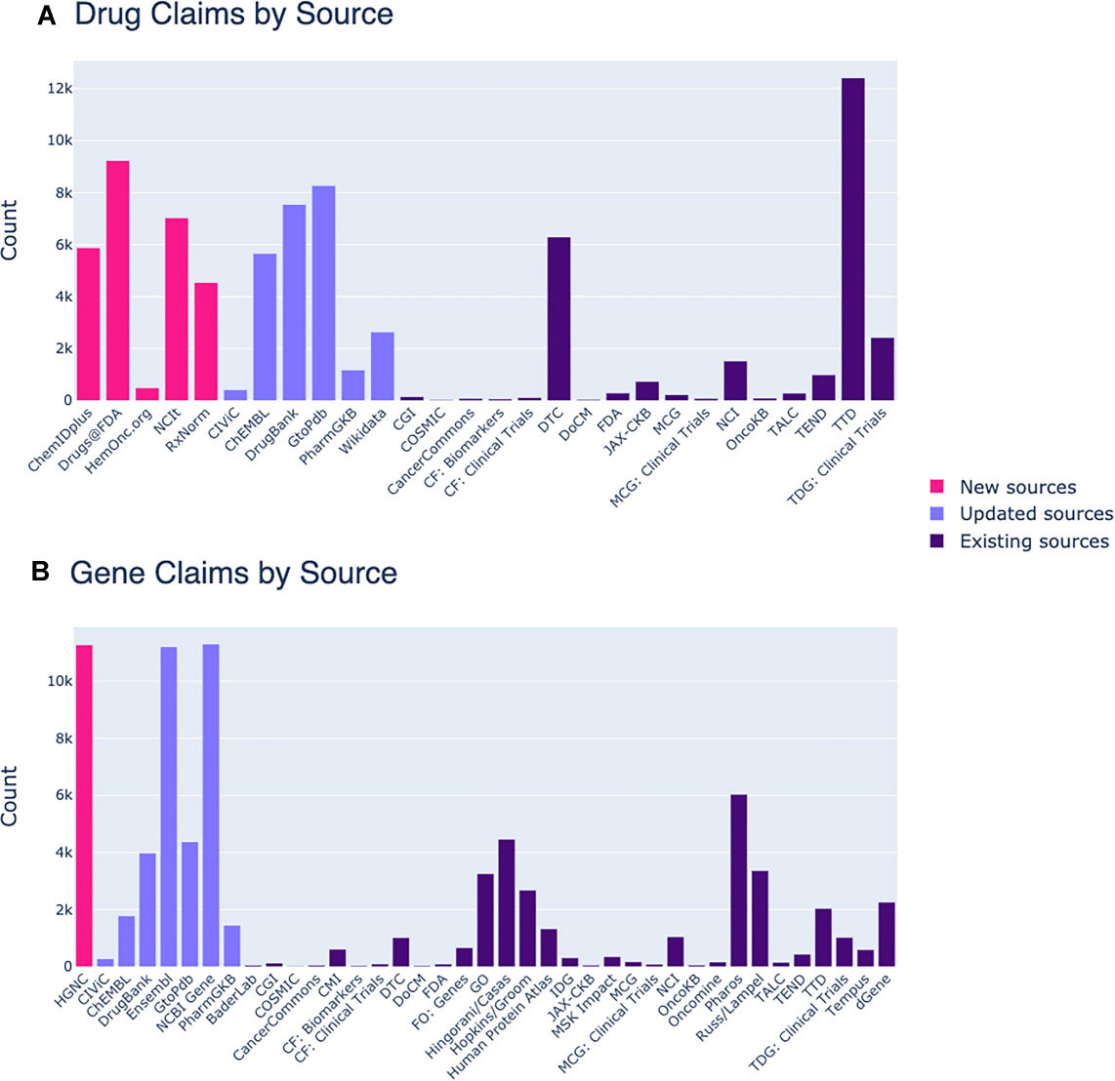
New and updated sources in DGIdb v5.0. Several new sources have been added to DGIdb and many existing sources have been updated. (**A**) Five new drug claim sources have been added and many existing sources updated. New drug claim sources include ChemIDplus, Drugs@FDA, HemOnc, NCIt and RxNorm. (**B**) HGNC has been added as a new gene claim source and many existing sources have been updated. Abbreviations: CF, Clearity Foundation; CGI, Cancer Genome Interpreter; CIViC, Clinical Interpretation of Variants in Cancer; DoCM, Database of Curated Mutations; DTC, Drug Target Commons; FDA, Food and Drug Administration; FO, Foundation One; HGNC, HUGO Gene Nomenclature Committee; IDG, Illuminating the Druggable Genome; JAX-CKB, The Jackson Laboratory Clinical Knowledgebase; MCG, My Cancer Genome; MSK, Memorial Sloan Kettering; NCI, National Cancer Institute; NCIt, National Cancer Institute Thesaurus; TALC, Targeted Agents in Lung Cancer; TDG, The Druggable Genome Clinical Trial; TEND, Trends in the Exploration of Novel Drug Targets; TTD, Therapeutic Target Database.

**Figure 3.**
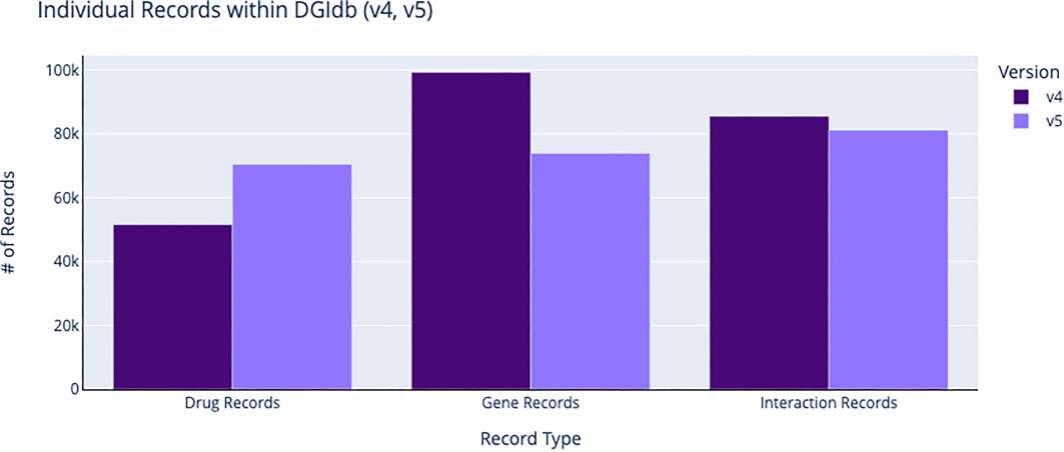
Record counts for drug, gene and interaction claims within DGIdb (v4.0 compared against v5.0). The number of individual records for each record type (drug, gene, interaction) is shown. The number of drug records has increased, while records for genes and interactions have been trimmed to just normalized records, resulting in an increase in data quality for the end user.

We have added new data and updated many existing sources for version 5.0. Six new sources have been added to DGIdb for drug and gene claims (Figure 2). Five of six are new drug claim sources and include ChemIDplus (10), Drugs@FDA, HemOnc (11), NCIt (National Cancer Institute Thesaurus) (12) and RxNorm (13). Three of these sources (ChemIDplus, HemOnc, NCIt) are structured vocabularies for drugs and therapeutics, and contain myriad synonyms and aliases to support drug claims and improve drug concept grouping. The incorporation of HemOnc in particular supports the capture of expert-curated knowledge for therapeutics for the fields of hematology and oncology, thus increasing the clinical utility for DGIdb within these domains. Interestingly, in the course of development of DGIdb v5.0, ChemIDplus was retired by the National Library of Medicine in favor of focusing development on PubChem (14) as their primary source of chemical information. We found that the ChemIDplus data set was useful for linking claims during the grouping step and therefore included this source despite retirement. Continued support of this data set into DGIdb will be invaluable for linking ChemIDplus identifiers utilized across historical literature. Two of the new sources (RxNorm and Drugs@FDA) contain curated data regarding chemicals and therapeutics that have pharmaceutical applications or a history of prior use in humans. In particular, Drugs@FDA was included to associate drug records with regulatory approval status. In prior versions of DGIdb, we found that reported regulatory information for our drug records could conflict with each other due to existing geopolitical and regulatory bodies for each source. While regulatory bodies can differ in their approval status of certain therapeutics, in aggregate it was possible to retrieve confounding approval classifications for the same drug (e.g. ‘approved’ versus ‘discontinued’). With this update, we chose to include Drugs@FDA as a new source to associate existing drug records with new drug applications (NDAs) and abbreviated new drug applications (ANDAs), thus further supplementing approval status with active market products [and their Food and Drug Administration (FDA)-approved labeling] for users who preferentially look to the FDA for regulatory status. Taken together, these sources have added an additional 27 172 drug claims to the database (Figure 3).

To support additional gene claims and harmonization within DGIdb, the HUGO Gene Nomenclature Committee (HGNC) protein-coding gene set (15) was added as an additional source. The HGNC has historically been responsible for approving unique symbols and nomenclature used to describe genes and the human genome. Including this source added an additional 11 272 gene claims to the database and supported improved gene concept grouping for existing gene claims within DGIdb (Figure 3).

In addition to these new sources, many existing sources for drug, gene and interaction claims have been updated to their latest version (Table 1 and Figure 2). The Clinical Interpretation of Variants in Cancer (CIViC) knowledgebase (16–18) was updated to its latest nightly release (as of June 2023) and includes all up-to-date drug, gene and interaction claims. Similarly, the IUPHAR/BPS Guide to Pharmacology (19) has been updated to version 2023.1 and PharmGKB (20) has been updated to its latest release (as of June 2023). This update has brought ChEMBL up to version 32 in DGIdb (previously version 27). Added in our previous major update (4.0), Wikidata (8) has been updated to a 2023 release (as of May 2023) and adds additional drug records to DGIdb. Ensembl (21) gene sets have been updated to their latest version (Ensembl 109) for this update.

**Table 1.** Additional source updates for DGIdb

Source	Status	Version	Records imported
ChemIDplus	New	2/22/23	D
HemOnc	New	44923	D
NCIt	New	23.04d	D
Drugs@FDA	New	Latest^†^	D
HGNC	New	Latest^†^	G
RxNorm	New	Latest^†^	D
ChEMBL	Updated	2/1/00	D, G, I
Ensembl	Updated	109	G
Guide to Pharmacology	Updated	7/15/05	D, G, I
CIViC	Updated	Latest*	D, G, I
PharmGKB	Updated	Latest*	D, G, I
Wikidata	Updated	Latest^†^	D

The asterisk denotes latest version as of June 2023. The dagger symbol denotes latest version as of May 2023. Types of records imported include drugs (D), genes (G) and interactions (I).

### New drug and gene grouping improvements (with concept IDs)

**Figure 4.**
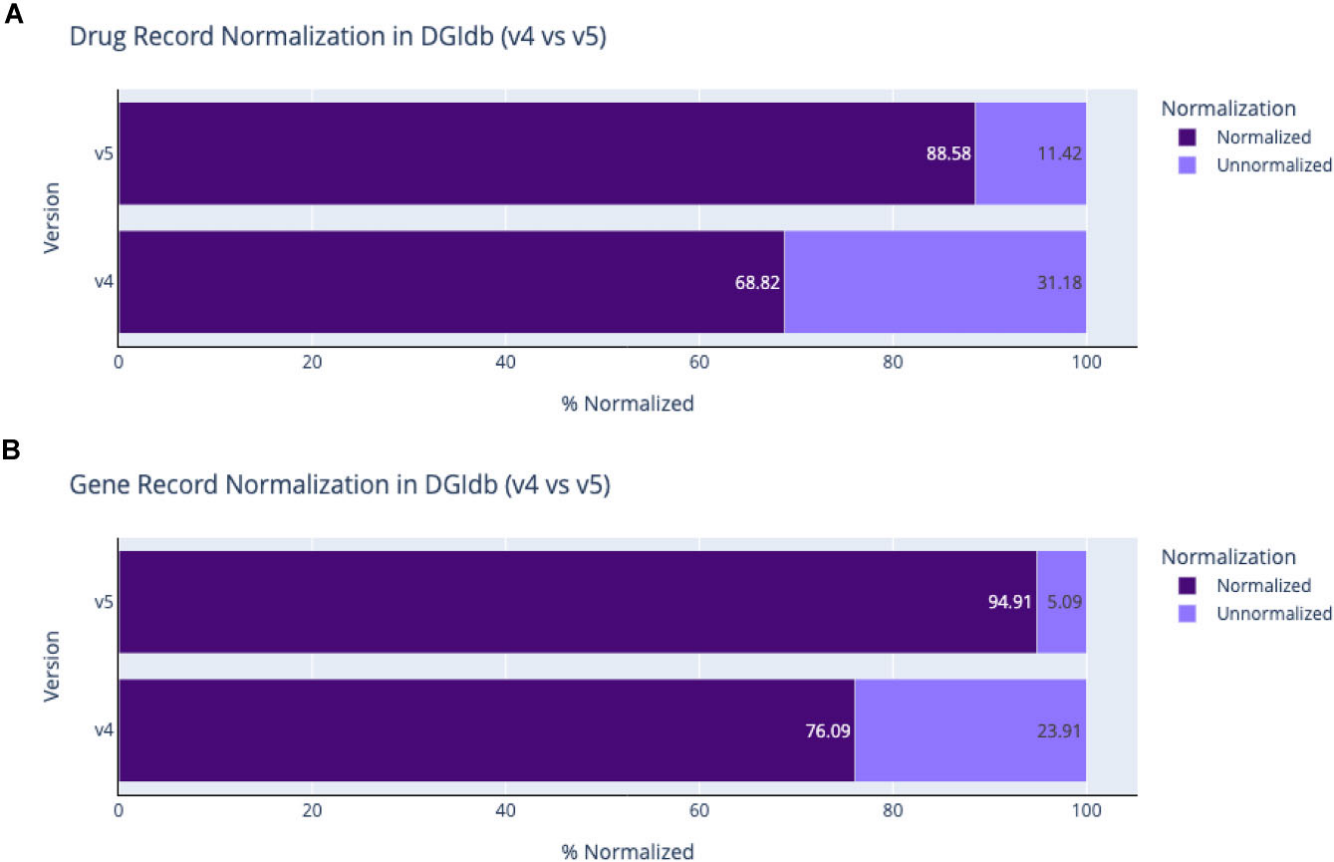
Normalization updates for drug and gene records across DGIdb (v4.0 compared to v5). (**A**) Drug normalization using thera-Py shows improvement in rates of normalization from 68.82% of records in v4.0 to 88.58% of records in v5.0. (**B**) Gene normalization using the VICC Gene Normalizer shows improvements in rates of record normalization of 76.09% in v4.0 to 94.91% in v5.0.

Changes have also been made to grouping methods within DGIdb. All drug and gene records are now normalized via modular normalization services as part of the import process (Supplementary Figure S1). This change has led to an increase in both normalized drug and gene concepts (Figures 3 and 4), resulting in an increase in data quality for the end user. The notion of concept grouping for drugs was first introduced in DGIdb v2.0. Follow-up versions of the database refined the grouping process, eventually utilizing the Variant Interpretation for Cancer Consortium’s (VICC) normalization service (https://github.com/cancervariants/therapy-normalization). This service normalized imported drug claims to matching ChEMBL and Wikidata records using a priority match search against primary labels and aliases. Since its original implementation within DGIdb, this therapy normalization service has undergone numerous changes. Most recently, it has been updated to utilize a graph-directed approach to follow explicit, human-curated references across databases to establish normalized therapeutic concept groups. In this latest release, we utilize improvements to the normalization service (now named thera-Py, https://go.osu.edu/TPY) to extend the coverage of our drug harmonization from 68.82% of imported drug claims in v4.0 to 88.58% of drug claims in v5.0 (Figures 3 and 4). Similarly, by utilizing improvements to the VICC gene normalization service (https://github.com/cancervariants/gene-normalization), we have improved gene normalization rates from 76.09% of gene claims in v4.0 to 94.91% of gene claims in v5.0. Notably, due to this change in grouping methodology, many existing claims from Ensembl that were not previously searchable have been dropped. Data from Ensembl are imported through the gene normalizer, resulting in the import of only those claims that have a normalized gene associated with them. While fewer gene claims are imported overall, there are now more searchable and well-described gene claims within DGIdb (Supplementary Figure S1).

### GraphQL query language in DGIdb

The introduction of GraphQL into DGIdb has allowed for the creation of *ad hoc* queries to retrieve drug–gene interactions as well as any additional linked data for use in downstream clinical and drug discovery applications. GraphQL is a query language that supports broad queries by utilizing highly described connections between data to identify and extract connected data points within a database. By utilizing edges connected to nodes of interest, any and all data points of interest for any specific starting node can be quickly and easily retrieved. GraphQL has been explored in recent years for potential applications in improving interoperability across healthcare management and information systems (22–24). We chose to add GraphQL as an additional abstraction layer on top of DGIdb to support interoperability with new and existing clinical variant interpretation and drug discovery pipelines. In applying GraphQL within DGIdb, we can implement normalized drugs or normalized genes as types of nodes and follow their edges to quickly identify interactions, as well as all associated druggability and functionality annotations, regulatory approval classifications, active ANDA/NDAs, sourcing, publications and attributes for either the interaction or the linked drugs and genes. By reformatting the database in this manner, clients are now able to request the underlying data in any configuration or format that fits their existing application. This enables various types of pipelines with differing needs to be more precise and efficient in how they access and format the data, whether they be clinical research, drug discovery or otherwise.

**Figure 5.**
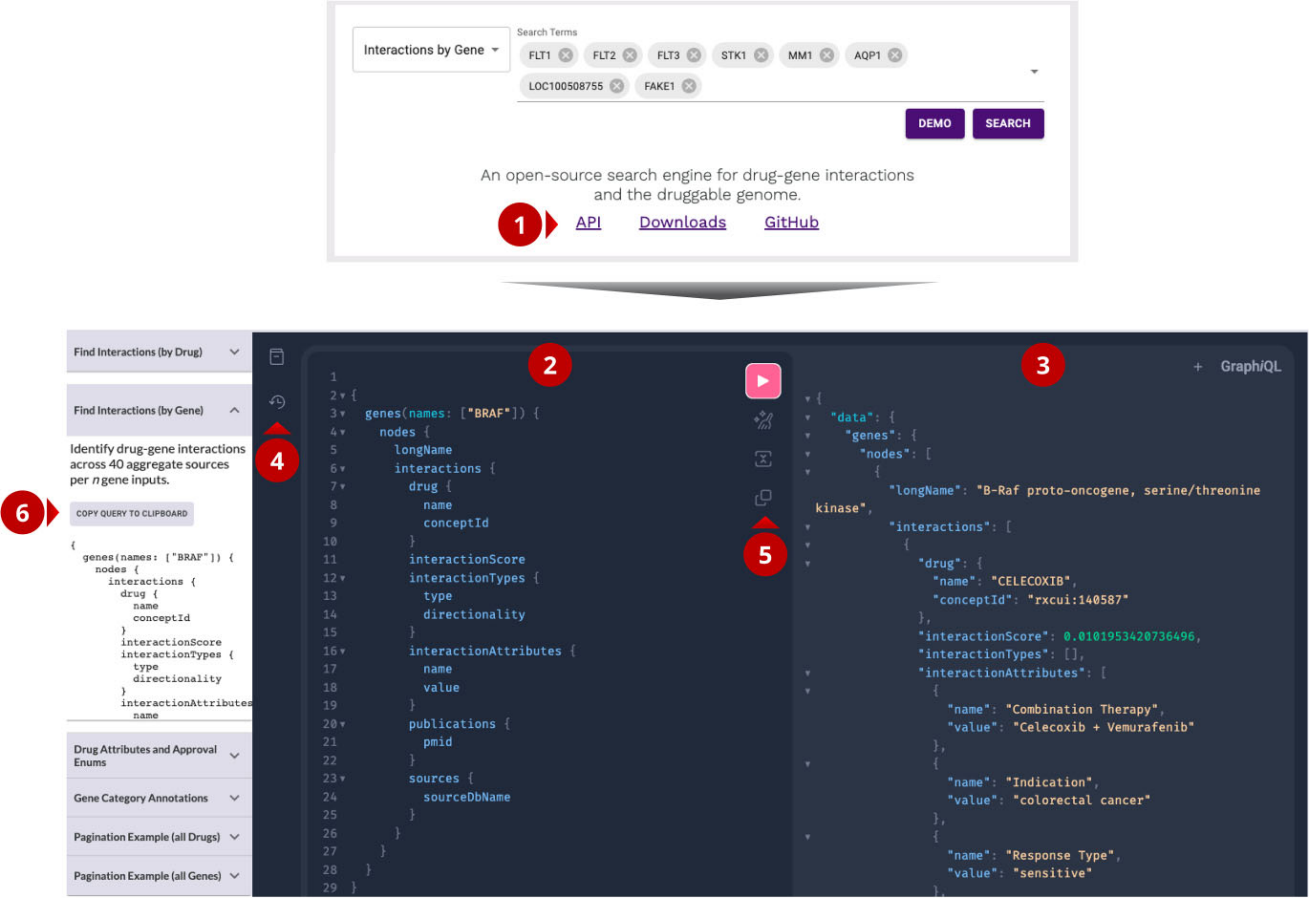
Addition of GraphQL enables *ad hoc* queries in DGIdb. GraphQL playground interface and example queries enable users to write their own custom queries for aggregate interaction and categories data. (**1**) To access the GraphQL API playground page, click the API footer button on the main page. (**2**) GraphQL query panel. All queries to be run can be written and edited within this panel. (**3**) GraphQL results panel. Results from queries written in panel 2 will populate here. (**4**) Documentation and query history panel. Documentation for all supported query types and data points can be accessed from here. Similarly, past queries are saved and can be rerun as needed. (**5**) Query control functionality. Once written, queries can be executed, prettified and merged using the buttons available. (**6**) Demo queries panel. Example queries are provided for commonly used search types. Queries can be copied into panel 2 and run as written, or edited further for additional data points. Additional examples of *ad hoc* queries have been provided in Supplementary Figures S2 and S3.

With the addition of GraphQL to DGIdb, a new API playground page has been introduced to allow users to learn how to write and test custom queries (Figure 5). This page can be reached by following the ‘API’ link in the footer of the main page. In the center of the API page is the playground query and response panel. Once written, queries can be executed using the ‘execute’ button in the center of the panel (Figure 5, panel 5). Example queries corresponding to commonly used search types within DGIdb have been provided on this page (Figure 5, panel 6) and additional examples of *ad hoc* queries are provided in Supplementary Figures S2 and S3. These queries can be edited further or run as is within the playground page itself, or externally in a formatted API call using a preferred programming language.

### New frontend interface

**Figure 6.**
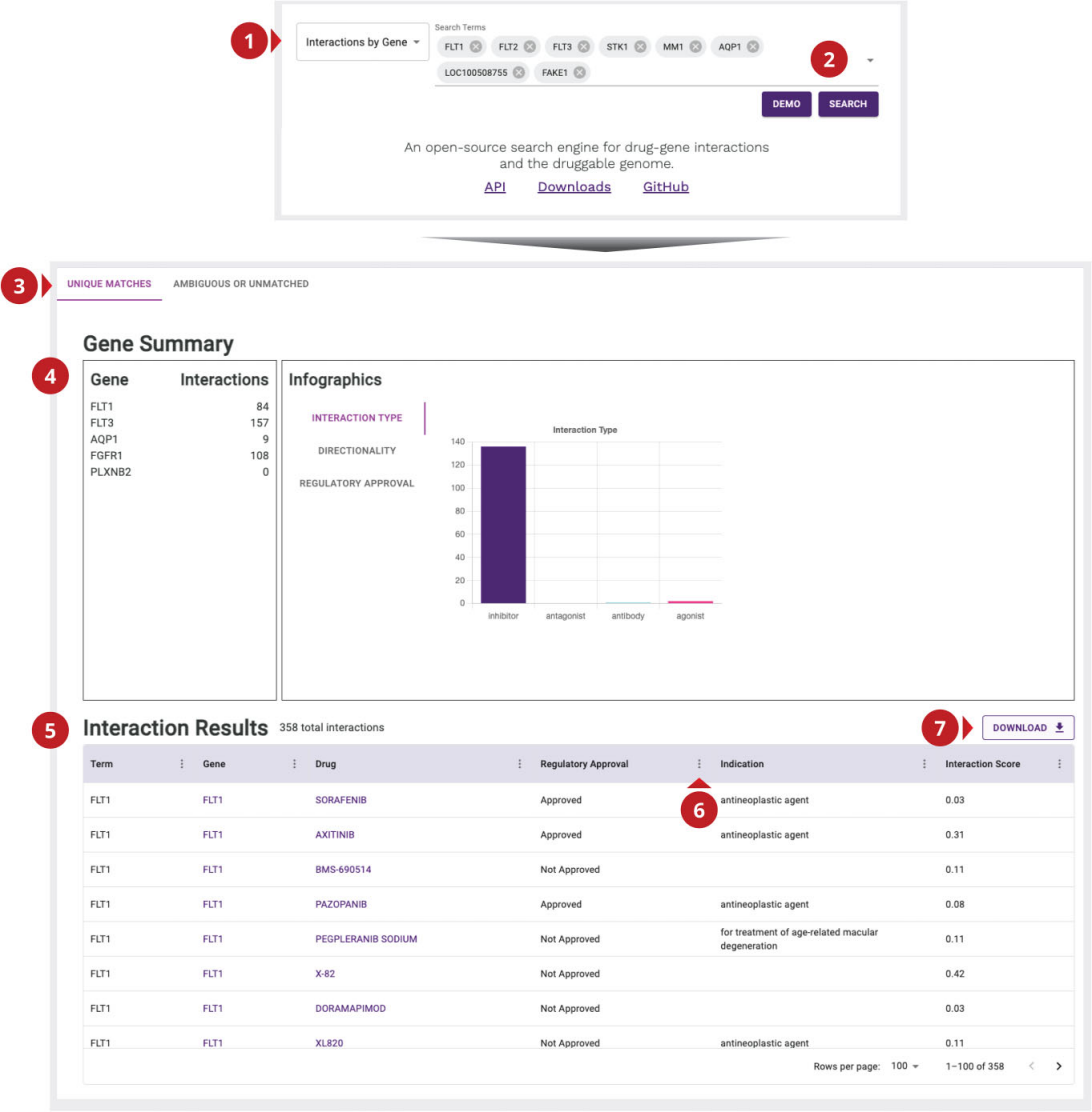
New UI expands search and filter control for interaction searches. Implementation of ReactJS into DGIdb has enabled a complete redesign of existing UI elements. (**1**) Search type selection. Desired search type can be selected from the main page dropdown: interactions by gene, interactions by drug and gene categories. (**2**) Search field. Once all drugs or genes of interest are entered, the search button can be used to submit the search request. (**3**) Match type tabs. All unique matches for requested terms will populate the ‘Unique Matches’ tab. Terms that are ambiguous (have multiple potential matches) or do not return any results will populate the ‘Ambiguous or Unmatched’ tab. (**4**) Gene summary panel. A summary of unique results for each entered search term will populate this panel. ‘At-a-glance’ infographics are provided for different metrics of the result set. Individual search term labels can be selected to filter both the infographics and results table. (**5**) Interaction results table. Unique results for searched terms will populate this table. Results contain all: searched term, gene name, drug name, regulatory approval status, known indications and interaction score. This table can be filtered using the search terms in panel 5 or using additional filter controls shown in panel 6. (**6**) Additional filter controls. Dropdowns on each column in the results table can be filtered with additional options. These controls allow filtering for specific strings of interest using common relational operators, such as ‘contains’, ‘equals’, ‘starts with’, ‘ends with’, ‘is empty’, ‘is not empty’ and ‘is any of’. (**7**) Download results. Results from search can be downloaded and analyzed externally from DGIdb. Results are downloaded in a TSV format.

With the switch to the React library, we have taken the opportunity to redesign DGIdb’s HTML user interface for a more intuitive experience (Figure 6). In brief, search pages from our previous version have been consolidated into a unified landing page with design features familiar to modern websites. Upon successful search, the landing page transitions seamlessly into a comprehensive results page. The interaction results pages from our previous version have been consolidated into a single results table with optional filtering capabilities and summary visualizations to facilitate dynamic inspection of data.

The new interface changes are available for use at https://dgidb.org (Figure 6). From the main page, the desired search type can be selected from the dropdown (Figure 6, panel 1). Similar to previous versions, the current supported search types are interactions by gene, interactions by drug and gene categories. Search terms of interest can next be entered into the corresponding search bar and executed by following the ‘Search’ button. The results page will be populated, with unique matches populating the ‘Unique Matches’ tab and all unmatched or ambiguous results (terms with more than one possible mapping) populating the ‘Ambiguous or Unmatched’ results tab. The results page contains two new components: the summary panel (Figure 6, panel 4) and the interaction results table (Figure 6, panel 5). The summary panel contains the list of all matching terms and three ‘at-a-glance’ visualizations for the interaction set. These can also be used to filter the data within the interaction results table. This table contains all unique matches for the search terms, as well as additional clinically relevant data points. These contain searched term, matched gene name, matched drug name, regulatory approval status, known indications (if available) and interaction score. Drug and gene names within this table can be clicked to navigate to individual record pages containing aggregate data. These include aliases, category annotations (for genes), approval values and active ANDAs/NDAs (for drugs), publications, known interactions (as well as associated PMIDs and sourcing) and all imported record attributes. Rows within the interaction results table can be clicked as well to retrieve individual interaction record pages. These pages contain details on the interaction partners, associated publications and all imported interaction attributes. These results can be filtered using the summary panel, or by using additional filter settings present within each column dropdown. These controls allow for filtering based on string relational operators. Available operators are ‘contains’, ‘equals’, ‘starts with’, ‘ends with’, ‘is empty’, ‘is not empty’ and ‘is any of’. Lastly, these data can be downloaded in a TSV format for external analysis using the download button (Figure 6, panel 7).

## Summary and future directions

DGIdb has received significant updates with this 5.0 release. Six new data sources have been added to DGIdb and eight existing sources for drugs and genes have been updated. Similarly, grouping methods for drug and gene claims have been improved. Drug and gene claims are now normalized via external normalization searches during import. These changes result in more searchable, higher quality data. The existing Ruby on Rails architecture for DGIdb was divided into separate server and client components. The server component remains a Ruby on Rails platform supporting a PostgreSQL database; however, a GraphQL communication layer has been inserted. As a result, existing data importers have been modified or rewritten and GraphQL-specific relation definitions have been added to the data model. The client component of DGIdb is now a ReactJS-driven interface with two primary methods for accessing the data. The HTML interface has been completely redesigned and many previous UI elements have been consolidated. The addition of GraphQL to DGIdb has allowed for a new endpoint that utilizes GraphQL query language to write *ad hoc* queries. These changes to architecture have been made with the goal of rebuilding DGIdb for seamless integration into existing clinical and drug discovery pipelines.

While these changes to DGIdb have improved content, access and usability for DGIdb, there is room for further improvement and iteration. With the modularization of DGIdb’s client component, we would like to develop tooling to support user curation and evidence submission features. By allowing users to submit and approve interaction evidence, DGIdb’s available data set would continually be up to date with the state of the field. One of the main goals of this update was to rebuild DGIdb for clinical and drug discovery pipelines. With that in mind, there are further refinements to the DGIdb data model that could be improved to better support these pipelines. Many imported data points associated with interactions are stored as simple key–value pairs within the singular ‘interaction attribute’ header. For example, many interactions between genes and anticancer agents have associated attributes for ‘Response Type’, ‘Combination Therapy’ or even ‘Alteration’. Formalization of these data points within the DGIdb data model would increase their visibility for our user base and further increase our clinical utility. In addition to refining the data model, we would also like to provide additional protection for users against false or retracted information. While we have performed an analysis utilizing the retraction watch database demonstrating a low incorporation of retracted PMIDs (24 PMIDs, 0.196%) (Supplementary Figure S4), we are exploring additional methods to flag retracted data. Finally, we would like to expand DGIdb and build AI-assisted tooling that would allow us to automatically create tentative interaction claims within DGIdb from additional definitive sources. Once created, these tentative interaction claims could be approved by field experts and become searchable within DGIdb, further expanding our searchable data set for our users. Taken together, these changes would allow DGIdb to further become an essential component of clinical and drug development pipelines.

## Supplementary Material

gkad1040_Supplemental_File

## Data Availability

DGIdb is an open access database and web interface (https://dgidb.org) with open source code available on GitHub (https://github.com/dgidb/dgidb-v5) and Zenodo (https://doi.org/10.5281/zenodo.10027075) under the MIT license. We also provide data downloads for drug claims, gene claims and interaction claims on the website in addition to an SQL data dump (https://dgidb.org/downloads). Documentation about the API and its endpoints can be found on the website (https://dgidb.org/api). We aggregate content from 44 sources with varying content licenses. We provide users with licensing information on a source-by-source basis for content aggregated by DGIdb through our source information page available from https://dgidb.org/browse/sources.
