# Supplementary material for: DGIdb 5.0: rebuilding the drug–gene interaction database for precision medicine and drug discovery platforms
Source: Nucleic Acids Res. 2023 Nov 11;52(D1):D1227–35. doi: 10.1093/nar/gkad1040 (PMC10767982; doi:10.1093/nar/gkad1040)
Supplement: gkad1040_Supplemental_File [file gkad1040_supplemental_file.pdf]

SUPPLEMENTAL FIGURES

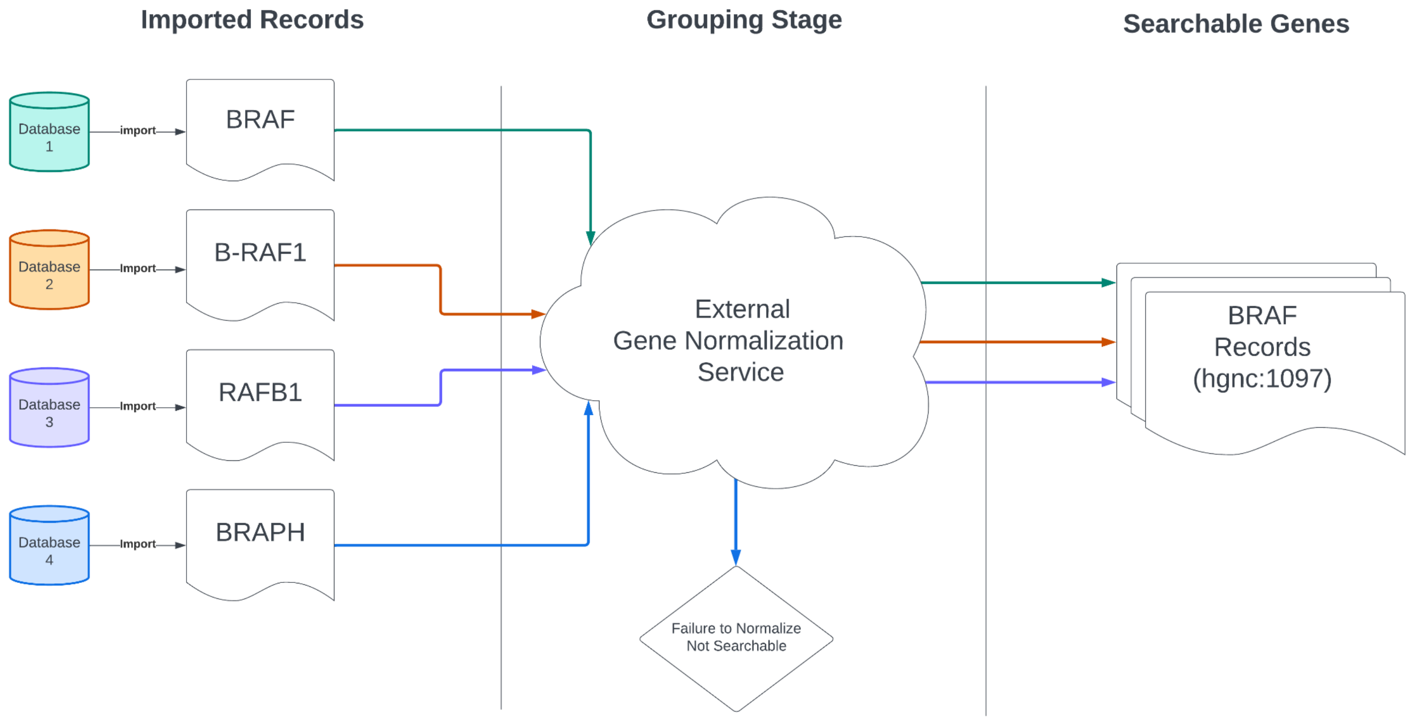

Supplemental Figure 1. **Example of Normalization Workflow.** Records for drugs and genes are first imported into DGIdb from aggregate sources. Individual records are passed through external normalization services to harmonize concepts during the grouping step. Records that successfully normalize are assigned an identifier corresponding to their gene or therapeutic concept and are considered searchable within DGIdb. Records that do not normalize are not assigned this identifier and thus are not searchable within DGIdb.

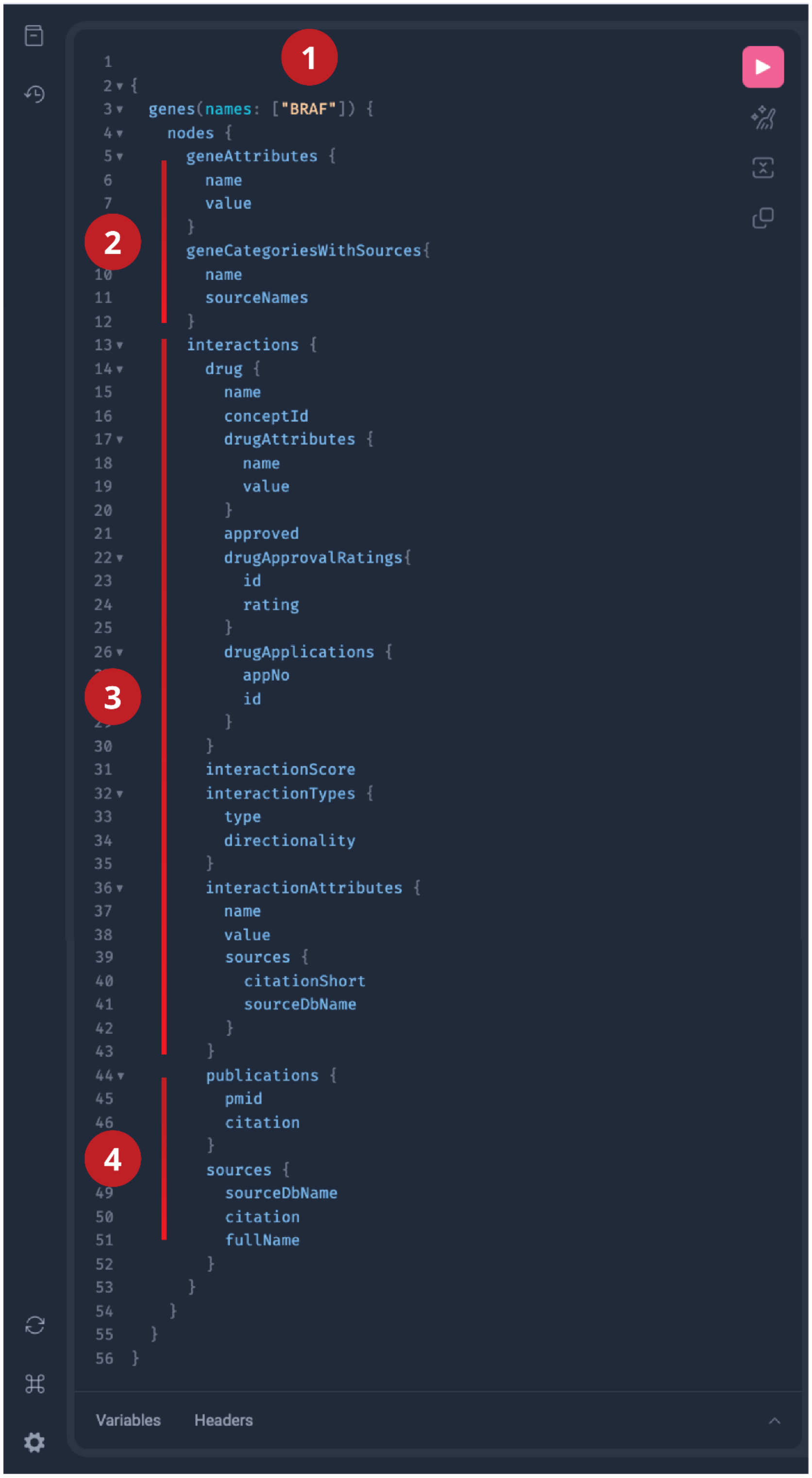

Supplemental Figure 2. Example of an *ad hoc* query for BRAF in DGIdb. Implementation of GraphQL into DGIdb allows for users to write their own custom queries for aggregate data. Provided is an example query for the gene ‘BRAF’ that has been expanded with additional nodes to bring in all interaction, category, and sourcing data. **(1)** A query for genes can be initiated by providing a list of gene symbols to the “names” field. This query supports multiple, comma-separated gene symbols. **(2)** All information related to the gene ‘BRAF’, including gene attributes and gene categories will be returned. **(3)** All data regarding interactions involving ‘BRAF’ will be returned. These data include all

drugs (and attributes for each drug), regulatory approval values, FDA applications, and interaction attributes (as well as attribute-specific sources). **(4)** All associated sources and publications for interaction data will be returned

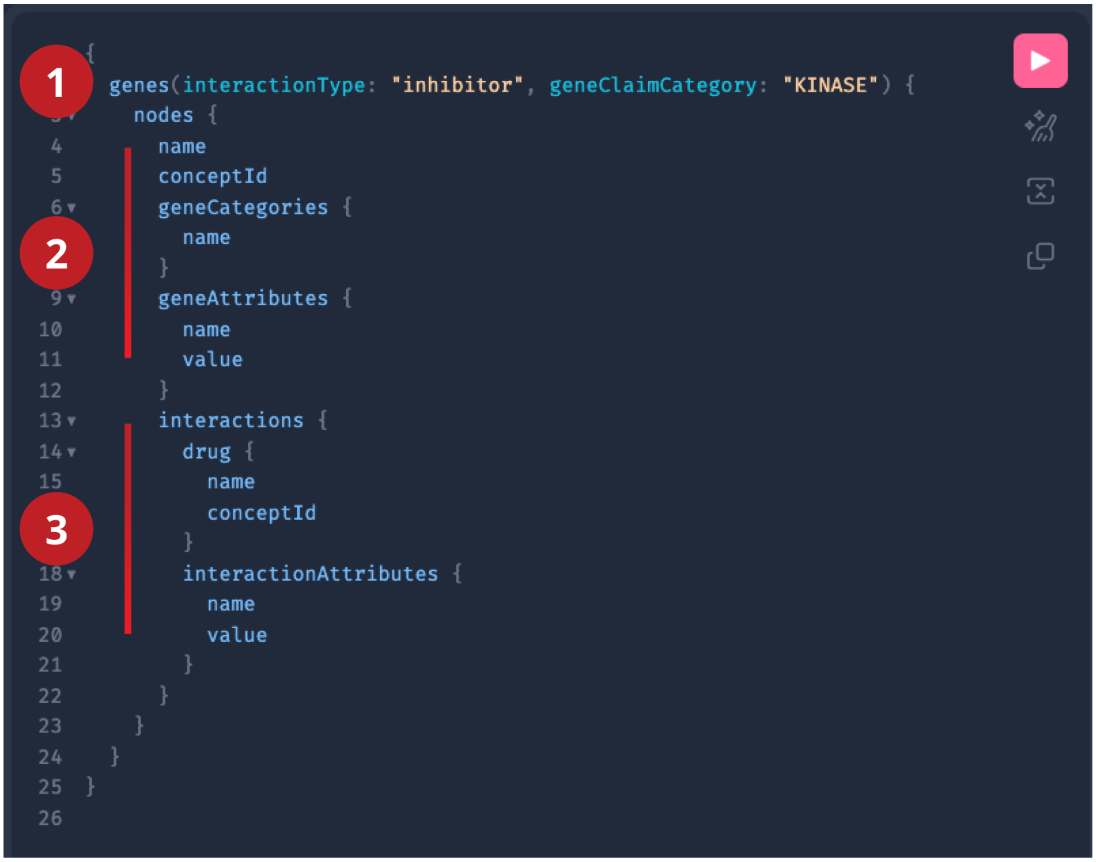

Supplemental Figure 3. Example of an *ad hoc* query for all kinase genes with an inhibitory reaction in DGIdb. Implementation of GraphQL in DGIdb allows for users to write precise queries for only genes matching desired specifications. Provided is an example query that returns all categories, attributes and interaction data for kinase genes with inhibitory reactions. **(1)** A query for all genes of category ‘KINASE’ with interactions of type ‘INHIBITOR’ can be initiated using the corresponding fields. **(2)** All information related to the categories and attributes for matching genes will be returned. **(3)** All interaction and interaction attribute data for matching genes will be returned.

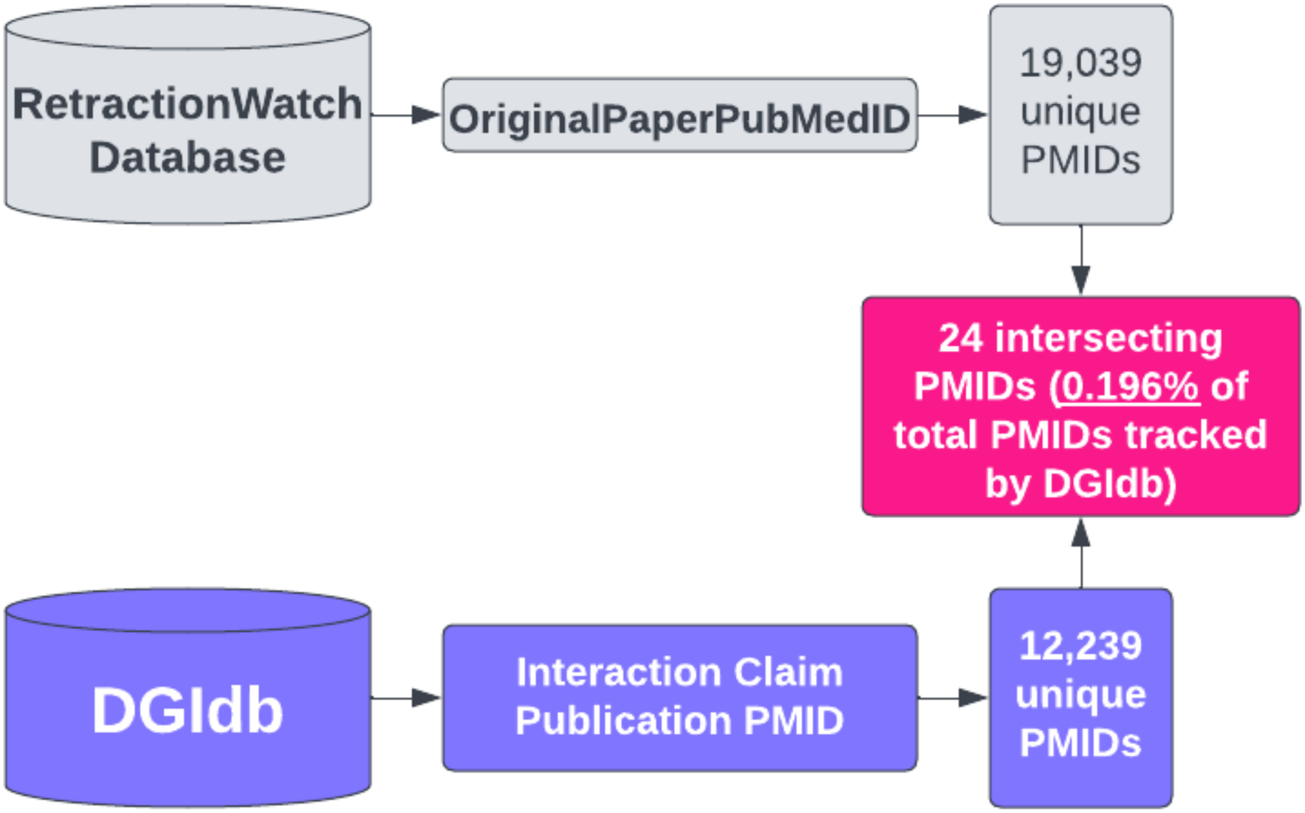

Supplemental Figure 4. Analysis of Retracted PMIDs in DGIdb. PMIDs associated with claims imported into DGIdb v5 were compared against PMIDs from known retracted papers taken from Retraction Watch DB. Of the 12,239 unique PMIDs within DGIdb's interaction data, only 24 PMIDs were found to be from retracted publications (0.196%).
